# Towards Whole-Body Fluorescence Imaging in Humans

**DOI:** 10.1371/journal.pone.0083749

**Published:** 2013-12-31

**Authors:** Sophie K. Piper, Christina Habermehl, Christoph H. Schmitz, Wolfgang M. Kuebler, Hellmuth Obrig, Jens Steinbrink, Jan Mehnert

**Affiliations:** 1 Department of Neurology, Charité University Medicine Berlin, Berlin, Germany; 2 Machine Learning Department, Berlin Institute of Technology, Berlin, Germany; 3 NIRx Medizintechnik, Berlin, Germany; 4 Institute of Physiology, Charité University Medicine Berlin, Berlin, Germany; 5 Department of Neurology, Max Planck Institute for Human Cognitive and Brain Sciences, Leipzig, Germany; 6 Clinic for Cognitive Neurology, University Hospital Leipzig, Leipzig, Germany; 7 Center for Stroke Research, Charité University Medicine Berlin, Berlin, Germany; Brigham and Women's Hospital, Harvard Medical School, United States of America

## Abstract

Dynamic near-infrared fluorescence (DNIF) whole-body imaging of small animals has become a popular tool in experimental biomedical research. In humans, however, the field of view has been limited to body parts, such as rheumatoid hands, diabetic feet or sentinel lymph nodes. Here we present a new whole-body DNIF-system suitable for adult subjects. We explored whether this system (i) allows dynamic whole-body fluorescence imaging and (ii) can detect modulations in skin perfusion. The non-specific fluorescent probe indocyanine green (ICG) was injected intravenously into two subjects, and fluorescence images were obtained at 5 Hz. The in- and out-flow kinetics of ICG have been shown to correlate with tissue perfusion. To validate the system, skin perfusion was modulated by warming and cooling distinct areas on the chest and the abdomen. Movies of fluorescence images show a bolus passage first in the face, then in the chest, abdomen and finally in the periphery (∼10, 15, 20 and 30 seconds, respectively). When skin perfusion is augmented by warming, bolus arrives about 5 seconds earlier than when the skin is cooled and perfusion decreased. Calculating bolus arrival times and spatial fitting of basis time courses extracted from different regions of interest allowed a mapping of local differences in subcutaneous skin perfusion. This experiment is the first to demonstrate the feasibility of whole-body dynamic fluorescence imaging in humans. Since the whole-body approach demonstrates sensitivity to circumscribed alterations in skinperfusion, it may be used to target autonomous changes in polyneuropathy and to screen for peripheral vascular diseases.

## Introduction

Whole-body fluorescence imaging is well established for small animal imaging in experimental biomedical research [1], [2]. Several contrast-enhancing biological mechanisms have been explored using a wide variety of more or less specific near-infrared (NIR) fluorescent contrast agents. Dynamic contrast-enhanced near-infrared fluorescence imaging (DNIF) allows a differentiation between tissue types and has been used extensively to detect blood flow dynamics [3]. The fluorescence dynamics measured have proved to be distinct in different organs [4].

The challenges of transferring whole-body fluorescence imaging to adult humans with comparably acceptable results in signal quality are manifold: (i) From being a matter of centimeters, the dimensions of the imaging object burgeon to over a meter and require a powerful, large-area excitation source and an extremely sensitive detection system. (ii) Penetration depth for the excitation light and the emitted fluorescence light is limited to the superficial tissue. (iii) The camera’s integration time must be restricted to sufficiently sample changes in fluorescence over time and thus measure fluorescence dynamics. (iv) Only a very limited number of fluorescent contrast agents have been approved for humans [5], [6]. As a consequence of i)–iv), fluorescence dynamics in humans have been studied only in body-parts. The dynamic absorption and fluorescence contrast of the unspecific blood-pool tracer indocyanine green (ICG) [7], [8] have been shown to detect signs of rheumatoid arthritis [9], hemodynamic changes in diabetic feet [10], sentinel lymph nodes and lymph drainage [11]–[15], and breast cancer [16]–[20]. Feasibility studies in the brain have demonstrated that even deep tissue can be targeted [21]–[24].

The successful translation of fluorescence-mediated molecular imaging to humans and the realization of their potential in diagnostics rely on having systems available which can scan the whole or large areas of the body [6], [12], [25].

The tissue penetration depth of NIR light suffices to trans-illuminate small animals but not humans. DNIF in humans will detect predominantly skin perfusion. Motivated by the results reported in rodents [4] and in human brain imaging [21]–[23], we focus in this study on the dynamics of bolusarrival when the whole body is scanned. More specifically we queried whether it is possible to detect the distinct perfusion properties of the highly perfused abdominal organs and whether the brain can be differentiated from the skin before ICG arrival in the skin. Furthermore, we modulated skin perfusion by varying temperatures to investigate how well differences in skin perfusion can be imaged.

## Methods

### Instrumentation

**Figure 1:**
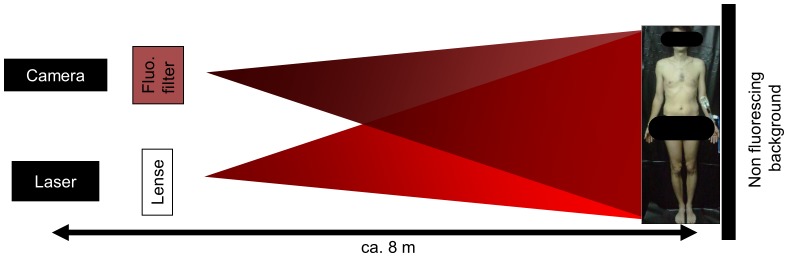
Schematic setup for whole-body fluorescence imaging in humans. Scheme of the experimental setup and photograph of subject 1 standing upright, ICG prepared for injection into the left cubital vein.

For illumination, the whole-body imaging set-up (Fig. 1) is equipped with a high-power 760 nm laser diode with integrated thermo-electric cooler (Intense Ltd., New Brunswick, NJ, USA) and 100 µm fiber pigtail. Total excitation power at the fiber output was approximately 200 mW, and a cylindrical lens was used to widen the laser beam. Illumination uniformity was optimized manually by adjusting the distance between laser output fiber and cylindrical lens to achieve maximal spreading of the laser beam throughout the target dimensions. Detection of the fluorescent light was accomplished by an ultra-sensitive and fast CCD camera (Evolve 512, Photometrics, Tuscon, AZ, USA, air cooled to −80°C) with the electron-multiplication gain set to its maximum of 1000. To image the subjects and collect the fluorescence light emitted, we used a focusing lens system (Nikkor macro lens, f = 28 mm, f/1.2, Nikon, Duesseldorf, Germany) and three combined 820 nm interference filters.

For the different measurements the camera integration time was varied between 170 and 500 ms (Table 1). The camera’s minimal internal storage time of 30 ms and acquisition time of 170 ms resulted in an overall, maximal imaging rate of 5 frames per second. Subjects were either standing upright or lying in supine position in a completely darkened room which was draped with non-fluorescent black sheeting. Figure 1 provides a sketch and a photograph of the experimental setup. Prior to fluorescence imaging, background images of the spatial excitation profile were made by imaging a plain, homogeneously fluorescing sheet for the correction of illumination inhomogeneities.

**Table 1:** Individual measurements and imaging setups.

Measurement# and day	Setup (distancecamera/laser –object)	Integration Time per image/totalnumber of acquired images	Subject N°	Subject position	Camera’s field of view
1 (day 1)	pilot setup (8 m)	500 ms/399	1	standing upright	wholebody
2 (day 2)	pilot setup (8 m)	200 ms/700	1	standing upright	wholebody
3 (day 2)	pilot setup (8 m)	200 ms/700	2	standing upright	wholebody
4 (day 3)	close-up (1.2 m)	200 ms/3180	2	supine position	abdomen
5 (day 3)	close-up (1.2 m)	200 ms/1590	2	supine position	head
6 (day 4)	Close-up, with hotand cold pads (2 m)	170 ms/3180	1	supine position	chest and abdomen
7 (day 4)	close-up, with hotand cold pads (2 m)	170 ms/3180	2	supine position	chest and abdomen
	Columns list the measurement day, the approximate distance between laser/camera and subject, which subject was imaged, the subject’s position and the field of view of the camera for all 7 individual measurements.				

### Ethics Statement

The procedure was in agreement with the Ethical Guidelines of the Institution and the Declaration of Helsinki. Subjects participated voluntarily and provided written informed consent prior to enrolment. Approval was obtained from the ethics committee of the Charité University Medicine Berlin (Nr.103 17.5.2000, Charité Campus Mitte, Charité University Medicine Berlin).

### Participants

The two principal investigators JS and HO (mean age 44) were imaged before, during and after bolus injection of the fluorescent contrast agent ICG (Pulsion AG, Munich, Germany). Subjects were healthy and did not have any known blood vessel related diseases. Subjects were examined 3 and 4 times, respectively. They have seen this manuscript and figures and have provided written informed consent, as outlined in the PLOS consent form, to publication of the manuscript and their photographs.

### Experimental Procedure

We performed a total of 7 bolus injections in three sessions. For each measurement, a single bolus of 25 mg ICG dissolved in 15 ml aqua *ad injectionem* was injected within approximately 5 seconds into the cubital vein. The reference time point t = 0 s was set to the beginning of bolus administration. Table 1 provides an overview over the individual measurements. We conducted three measurements with the subjects standing upright and the camera and laser about 8 m away to image the entire body.

Subsequent to whole body views, we took a focused look at the abdominal area and the head to see if distinct perfusion properties were discernible in the inner organs or the brain. We thus had two consecutive close-up measurements in subject N° 2 with a focused laser excitation and field of view of the camera: the first one on the abdomen (first ICG bolus) and then, one hour later, one on the head (second ICG bolus). Here, the distance between laser source/camera and subject was only about 1.2 m.

To modulate skin perfusion by temperature, two hot pads (∼40°C) and two cold pads (∼0°C) pads were placed for 5 minutes on the upper part of the body. All pads were removed just prior to ICG injection and fluorescence recording. For these measurements, the distance between laser source/camera and subject was about 2 m to facilitate a close-up field of view of the upper body.

Subjects received illumination with approximately 0.2 W/m^2^ during the whole-body measurements (illuminated area approx. 2×0.5 m^2^) and 0.8 W/m^2^ for the head and abdomen close-up measurements (illuminated area approx. 0.5×0.5 m^2^). This is well below the maximum permissible exposure limit of 2,000 W/m^2^ for skin at the used wavelength range and exposure duration according to the International Electrotechnical Commission standard (DIN EN 60825-1). Subjects were instructed to keep their eyes closed throughout the entire whole-body measurements and wore a non-fluorescent eye-protection in the close-up setups.

### Data Processing

The images were analyzed using Matlab software (The Mathworks, Inc., Natick, MA). Images were temporally and spatially filtered with a third order 0.5 Hz low-pass Butterworth filter and a two dimensional digital Gaussian filter (4 pixel diameter), respectively. Baseline subtraction was performed with baseline defined as mean value over the first 5 s after bolus administration. Spatial and temporal smoothing, which is necessary to diminish readout and thermal noise of the camera that originate from the required high amplification gain, was performed according to previous studies from our lab for planar NIRF imaging (Piper et al., 2010) or ICG-bolus dynamics (Habermehl et al., 2012).

For measurement 1 we used a normalized fluorescence of the subject taken 3 minutes after bolus injection to correct for illumination inhomogeneities. For all following measurements, each image was divided by a spatially smoothed and normalized background image (two dimensional digital Gaussian filters, 20 pixel diameter) of the spatial laser profile without the subject to correct for illumination inhomogeneities similar to previously published protocols [26], [27]. Figures S1 and S2 present an example of the correction process and an uncorrected time series of measurement 1. Following temporal and spatial filtering, and appropriate correction for background and baseline, fluorescence images were merged to a video with a frame rate of 5 frames per second. In Video S1, color coding of the fluorescence intensity (a.u.) is equally scaled for both subjects. After 40 and 90 seconds, video rate is increased to 25 and 50 frames per second, respectively, as displayed in the video subtitle.

To map differences in skin perfusion, ICG arrival time was calculated for each pixel. Arrival time was defined as the time to 50% of fluorescence maximum of the individual pixel time course. We further extracted mean time courses of ICG dynamics in six different regions of interest (ROIs) on the upper body and calculated the corresponding standard deviations for the fluorescence intensity over all ROI-pixels. ROIs were selected with a size of 45×65 pixels over warmed and cooled skin areas and with a size of 20×65 pixels over interjacent neutral areas, respectively. Two-sample two-tailed t-tests were performed to test the arrival times in all pixels within different ROIs against the alternative hypothesis that each two independent samples are derived from distributions with different means. A total of 6 tests at a 0.05/6 = 0.0083 Bonferroni-corrected significance level were performed.

In analogy to small animal DNIF imaging [4], we extracted the mean from six different seed ROIs (10×10 pixel each) over a three-minute-long interval and performed a non-negative least-squares fit to calculate spatially separate and color-coded areas with a dynamic behavior similar to that of the pre-selected seed ROIs. Pixels outside the subject were excluded from this analysis.

## Results

**Figure 2:**
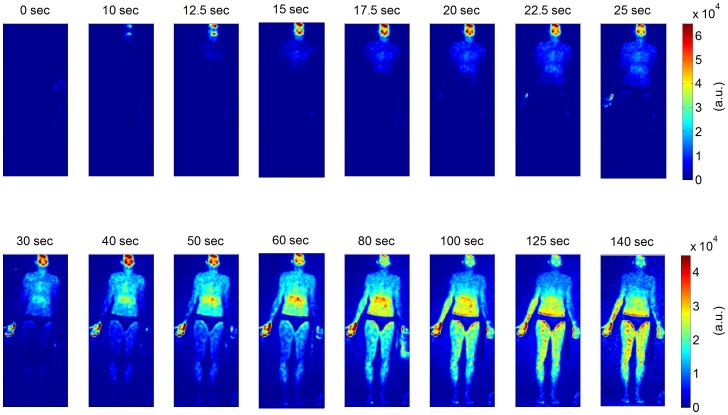
Whole-body fluorescence image time series in a healthysubject after intravenous bolus administration of ICG. Time zero seconds is set to the beginning of ICG injection. Time intervals between neighboring images differ. Color coding of fluorescence intensity in arbitrary units (a.u.) is equally scaled for neighboring images as indicated by the color bars on the right. Scaling is decreased in the bottom row.

Figure 2 shows the first whole-body fluorescence image time series in subject 1 (Table 1, measurement 1). It demonstrates the main dynamic features of bolus arrival across the whole-body. The bolus was injected at t = 0 s. After about 10 seconds, a first and very prominent fluorescent signal can be seen in the face, most prominent on the forehead, lips and cheeks. The chest and the abdomen show fluorescence about 15 seconds and 20–25 seconds after injection, respectively. After about half a minute, fluorescence from the legs can be detected, and after about 50 seconds there is a measurable signal from the feet. Since the excitation laser was largest in the centre of the camera’s field of view, the abdomen, thighs and right palm prior to correction show a much higher fluorescence signal than does the periphery (see also figure S2). This inhomogeneity is still discernible after image correction in the late stage (>60 s) fluorescence images of the same subject (Fig. 2).

In the following measurements images were corrected with a static planar background image of the spatial laser profile (see also figure S1). Video S1 shows an image sequence of background-corrected whole body fluorescence images demonstrating the flooding dynamics in two subjects (Table 1, measurement 2 and 3). Again, fluorescence can first be observed in the head (lips and forehead, ∼10 seconds post injection); it then spreads throughout the chest, abdomen, and the periphery. Fluorescence is most pronounced in the head (forehead, cheeks, and lips), the finger tips and the chest. The maximum signal intensities in the chest and the abdomen are reached after about 60 and 120 seconds, respectively, followed by a gradual decrease in fluorescence. The fluorescence partly exceeds the shape of the body because the fluorescence light is reflected at the wall behind the subjects.

**Figure 3:**
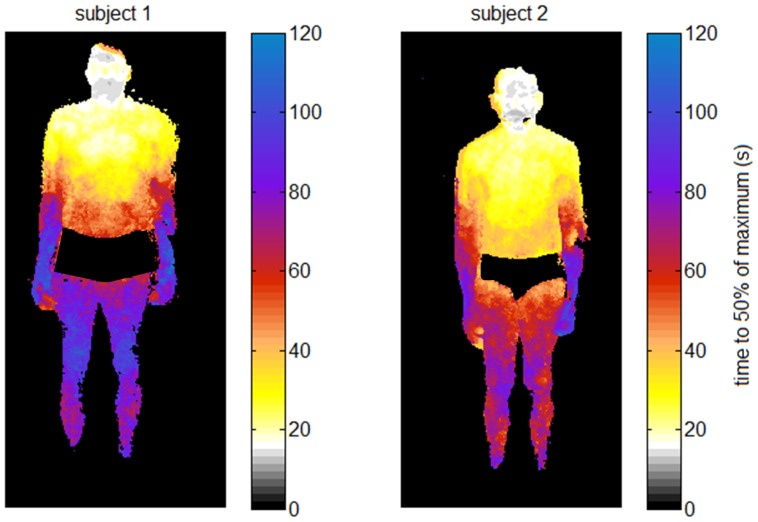
Mapping ICG arrival times over the entire body. The color of each pixel codes the point in time at which 50% of the maximum intensity of the pixel’s time course is reached.

Figure 3 shows the corresponding mapping of the ICG arrival times over the entire body for both subjects, calculated as the time to 50% of the maximum intensity. The map depicts the same dynamics, with first bolus arrival in the head (lips and forehead), then in the chest, abdomen and periphery. As expected in healthy subjects, arrival times are largely symmetrical over left and right body parts.

**Figure 4:**
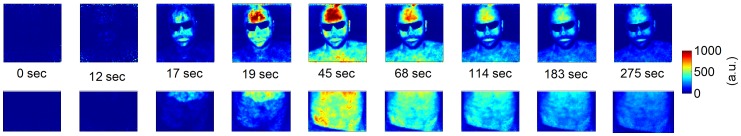
Close-up fluorescence image time series of the abdomen and the head. The subject received two consecutive intravenous bolus injections of 25’s field of view was on the abdomen. For the second bolus one hour later, the field of view was on the subject’s head. ICG bolus administration starts at time 0 seconds. Images are divided by a background image to correct for inhomogeneous distribution of the exciting laser light and baseline subtracted. Color coding of fluorescence intensity (a.u.) is equally scaled as indicated by the color bar except for the first two columns, wherecolor bar limits are [0 100].

The close-up measurements of the head and the abdomen (Table 1, measurements 4 and 5) allowed us to refine the presentation of fluorescence dynamics (Figure 4). In the face, fluorescence appears first on the lips and nose, followed by a prominent signal from the forehead. For the abdomen, fluorescence from the area of the respiratory muscle is prominent at the very beginning. Distinct perfusion properties of organs or the brain are not discernible with the close-up approach. However, the texture of the surface (ribs, navel and pelvic bones) is preserved, which shows that the technique provides basic anatomical information in the sub-centimeter range.

**Figure 5:**
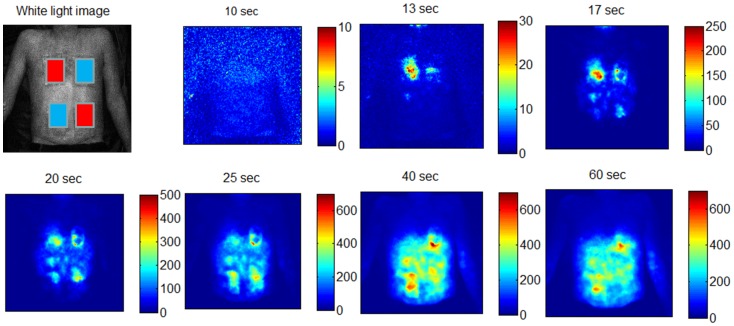
Fluorescence image time series after modulating local skin perfusion with heat and cold. Upper left: White light image of the subject’s upper body overlaid by the positions of the two warm and cold pads. Pads were placed on the upper body for 5 minutes and removed right before ICG bolus injection and fluorescence imaging started. While heat leads to an increase in skin perfusion due to auto-regulated vasodilation, releasing the cold pad leads to a protective vasodilator effect which is by comparison delayed. Color coding of fluorescence intensity (a.u.) is individually scaled for each image.

**Figure 6:**
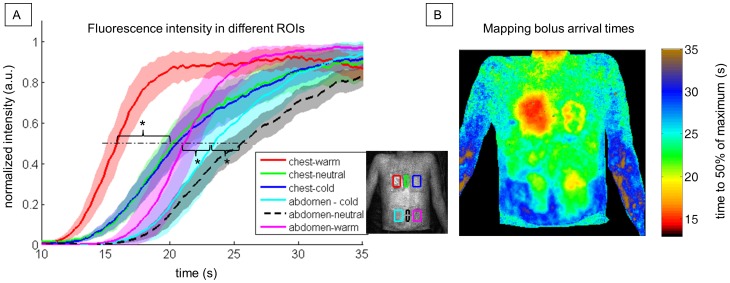
Fluorescence time courses and mapping bolus arrival times after modulating local skin perfusion with heat and cold. A: Normalized mean fluorescence time courses in six different regions of interest (ROIs) as indicated in the legend inlet. Shaded areas illustrate the respective intensity’s standard deviation (a.u.). The dot-dashed line indicates 50% of the maximum intensity, which was used to define the bolus arrival time. Asterisks (*) mark significant differences (p<0.0083) in bolus arrival times between warmed, cooled and neutral ROIs on chest and abdomen, respectively. B: Color-coded mapping of the time to 50% of maximum intensity within each pixel.

Figure 5 shows the fluorescence image time series after modulating local skin perfusion by heating and cooling. After about 13 seconds, there is a focal increase in fluorescence in the warmed chest area, whereas fluorescence is delayed for 5 seconds in the center of the cooled area. Interestingly, a ring-shaped perfusion penumbra can be observed between 17–25 seconds in the chest area, where the cold pad was placed. This might be caused by the local temperature gradient. After 17–20 seconds, ICG spreads throughout the abdomen, and the same delay effect is discernible for the warmed and cooled areas on the abdomen. Here, however, the contrast is not as prominent as in the chest. After a minute, cooled areas still show a prominent fluorescence signal. The fluorescence time courses extracted from six different ROIs (Figure 6A) and the color-coded map of bolus arrival times (Figure 6B) illustrate the temporal differences in ICG in-flow after local skin perfusion has been modulated: Warmed areas on chest and abdomen show a significantly faster bolus arrival compared to the corresponding neighboring cooled areas (p<0.0083). Bolus arrival times in the different ROIs (mean ± standard deviation) on the chest are 15.9±0.8 s, 20.2±0.9 s and 21±1 s for the warmed, neutral and cooled ROI, respectively. On the abdomen, corresponding bolus arrival times are 21±1 s, 26±1 s and 24±2 s, respectively. Here, also cooled and neutral tempered skin areas have significantly different arrival times (p<0.0083).

**Figure 7:**
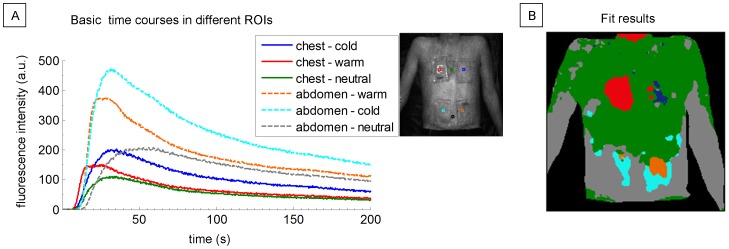
Color-coded mapping of areas with similar temporal behavior. A: Mean fluorescence basis time courses in six different regions of interest (ROIs) after modulating local skin perfusion with heat and cold on chest and abdomen. B: Color-coded fitting results mapping areas with temporal behavior similar to the extracted basis time courses calculated by a nonnegative least square fit.

Figure 7A shows the extracted fluorescence time courses from six small regions of the upper body corresponding to the center locations of the four pads and two neutral areas on the chest and abdomen, respectively. In Figure 7B the color coded results of a nonnegative least square fit are presented. Here, the areas with temporal behavior similar to the extracted basis time courses are equally colored, leading to a static map of separated regions with distinct skin perfusion. The shoulders and nearly the entire chest show the same fluorescence dynamics if not modulated by temperature. On the chest, the warmed area and the center of the cooled area are clearly separated from the surrounding skin. Abdomen and forearms also have similar fluorescence dynamics. The areas where the pads were placed on the abdomen are also distinguishable. Here, however, parts of the warmed area on the lower abdomen were fitted best by the temporal behavior of the cooled abdomen region (cyan colored).

## Discussion

To the best of our knowledge, this is the first report on whole-body dynamic fluorescence imaging in humans. The approach allows the non-invasive tracking of flooding dynamics of ICG across the whole body surface. A sub-centimeter spatial resolution and a high sampling rate (5 Hz) were achieved, allowing us to map local differences in skin perfusion.

In essence the whole-body DNIF set-up presented here is a translation of the well established approach in small animal imaging [3], [4], [25]. Notably our system has proven to be sensitive enough to image dynamic fluorescence for the increased object dimensions in humans. A sensitive detection system is mandatory: fluorescence was detected only with the electron-multiplication gain set to its maximum. On the other hand, even integration times of 170 ms rendered a sufficiently strong signal. Thus temporal resolution, one clear advantage of optical imaging, is preserved, as evidenced by a sampling frequency of about 5 Hz. This temporal resolution exceeds that of most other whole-body imaging modalities in humans such as positron emission tomography/computer tomography [28], [29], single-photon emission-computed tomography [30] or magnetic resonance imaging [31]–[33] in humans.

Because the illumination strength was strongest in the center of the camera’s field of view, correcting for inhomogeneous excitation is essential to analyze the data. This can be achieved by background correction of the resulting images [34]. Dividing each fluorescence image by a background image taken from a plain sheet might not be ideal because the spatial profile of the subjects includes elevations such as the nose and all curved body-parts. Illumination inhomogeneities might be best corrected using a background image of the subject illuminated with the excitation light and collected with the corresponding excitation wavelength filter [26], [27]. With our current camera set-up, rapid switching between excitation and fluorescence wavelength was not possible, but this could be realized in future applications with a filter wheel. Such an approach will enhance the specificity of differences detected in fluorescence.

When planar reflectance imaging is used, the intensity of the fluorescence detected correlates inversely with the depth of the fluorophore in the tissue [35]. While tissue penetration depth of NIR light suffices to trans-illuminate small animals, it does not suffice in adult humans, so the ICG signal will be dominated by changes in skin perfusion and superficial tissue layers. In the present study, distinct fluorescence patterns of inner organs or the brain were not discernible. When deep tissue such as the brain or the female breast is targeted, fiber-based diffuse optical tomography or time-resolved imaging systems seem mandatory [17], [18], [22], [36].

Nonetheless, DNIF imaging is suitable for detecting regional differences in skin perfusion with much smaller contributions from subcutaneous tissue. The perfusion maxima in tissues such as mucosa, finger tips, and the forehead are similar to regional differences described by infrared thermography [37], [38]. It should be born in mind that heat dissipation is primarily a function of skin perfusion. This nicely matches the fact that subcutaneous tissue is less vascularized and will not contribute strongly to the fluorescence signal described here. The mean arrival time of the fluorophore as detected in the facial skin within 10–20 seconds after bolus injection corresponds with previous data from our group demonstrating similar fluorophore kinetics for the extra-cerebral galeal tissue of the head in patients undergoing extracorporeal bypass, while kinetics for preferentially perfused inner organs such as the brain were markedly faster (<10 seconds) [24].

Our approach reliably detected regional differences in skin perfusion induced by cooling and heating the skin. In congruence with basic physiological principles of the thermal regulation of skin, perfusion was not only increased by heat stimulation, but also by release of cold stimulation, albeit with a delayed response. The latter is in line with the well-described delayed protective vasodilation effect following cold exposure [39] and could serve as a test for probing intact autonomous functions. The effects of heat and cold on the lower abdomen were thus attributed to the same dynamic fluorescence behavior (Figure 7B) because by the time ICG reached the skin of the lower abdomen, auto-vasodilation due to heat and the delayed protective vasodilation effect due to cold coincided.

Other groups have shown the usability of DNIF for detecting active rheumatoid arthritis in hands [9], vascular disorders in diabetic feet [10] and lymphatic disorders [11]. With the technology presented here, it is now possible to simultaneously image healthy tissue as a within-subject reference and scan entire bodies for vascular abnormalities. DNIF studies of hairy body regions, however, are largely limited as NIR light is highly absorbed by hair.

In our two healthy volunteers, ICG arrival times were distributed symmetrically between left and right body parts and showed an even flow in bolus arrival. On the other hand, in patients suffering from peripheral vascular disease, or polyneuropathy as is common in diabetes, micro-vascularization and regulation are locally compromised by the autonomous nervous system [40]. Using our approach, such pathological alterations could be detected as decreased or delayed fluorescence signals compared to healthy tissue. The fitting approach originally reported by Hillman and Moore [4] might be helpful in generating static maps of distinct skin perfusion properties and separating healthy from diseased areas of skin perfusion.

An alternative application may be the early identification of superficial cancers. These are characterized by higher vascularization and skin blood flow [41], and highly permeable blood vessels with extravasation and accumulation of ICG [14], [15], which in turn can be detected easily by DNIF.

If the analysis is not focused on relative within-subject variations but rather addresses quantitative differences across several subjects or measurement days, data normalization will be necessary. In this case, a fluorescent standard placed with each patient could account for inter-subject variability of the laser illumination and data could be expressed in target-to-background (standard) ratios at a particular point in time post injection.

Our results establish and demonstrate the feasibility of whole-body dynamic fluorescence imaging in humans. In addition, we provide evidence for its ability to non-invasively detect local differences in subcutaneous microcirculation. Future work may establish whole-body fluorescence imaging in humans as a screening tool of the wholebody or larger body parts for systemic pathologies, including lymphatic disorders, skin cancer, peripheral vascular disease, polyneuropathy or arthritis. In a next step, the diagnostic sensitivity and specificity of this novel and promising tool could be established in large scale clinical studies. Such studies will profit from the relatively undemanding setup, which can be extended to different protocols e.g. targeting extravasation of the contrast agent when using long intervals between injection and scanning.

## Supporting Information

Figure S1 **DNIF correction process.** A: Raw image taken 115 sec after bolus injection. B: Spatial smoothing with a two dimensional digital Gaussian filter (4 pixel diameter). C: Spatially smoothed and normalized background image of a plain light fluorescing sheet to correct for illumination inhomogeneities. D: Final image after background correction and baseline subtraction, defining the mean over the first 5 s after bolus administration as baseline. Color bars indicate fluorescence intensity in arbitrary units (a.u.).
(TIF)

Figure S2 **Whole-body fluorescence image time series of measurement 1 without illumination correction.** Time zero seconds is set to the beginning of ICG injection. Time intervals between neighboring images differ as indicated by image headers. Color coding of fluorescence intensity in arbitrary units (a.u.) is equally scaled for neighboring images as indicated by the color bars on the right.
(TIF)

Video S1 **Video: whole-body fluorescence in humans.** Image sequence of background-corrected whole-body fluorescence images that demonstrate the flooding dynamics in two healthy subjects. Color coding of the fluorescence intensity (a.u.) is equally scaled for both subjects. After 40 and 90 seconds video rate is increased as displayed in the video subtitle.
(AVI)
