# Supplementary figures and images for: Towards Whole-Body Fluorescence Imaging in Humans
Source: PLoS One. 2013 Dec 31;8(12):e83749. doi: 10.1371/journal.pone.0083749 (PMC3877082; doi:10.1371/journal.pone.0083749)

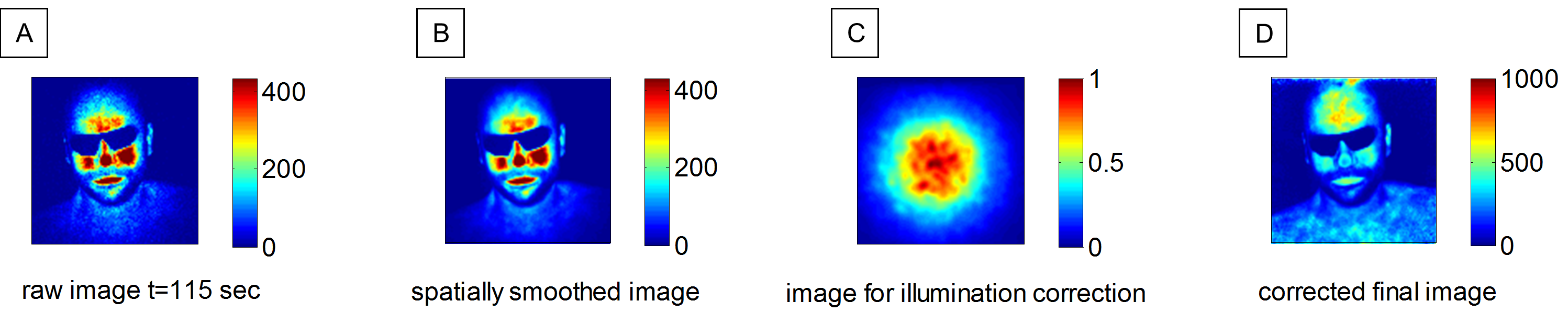

Supplement: Figure S1 — DNIF correction process. A: Raw image taken 115 sec after bolus injection. B: Spatial smoothing with a two dimensional digital Gaussian filter (4 pixel diameter). C: Spatially smoothed and normalized background image of a plain light fluorescing sheet to correct for illumination inhomogeneities. D: Final image after background correction and baseline subtraction, defining the mean over the first 5 s after bolus administration as baseline. Color bars indicate fluorescence intensity in arbitrary units (a.u.). (TIF) [file pone.0083749.s001.tif]

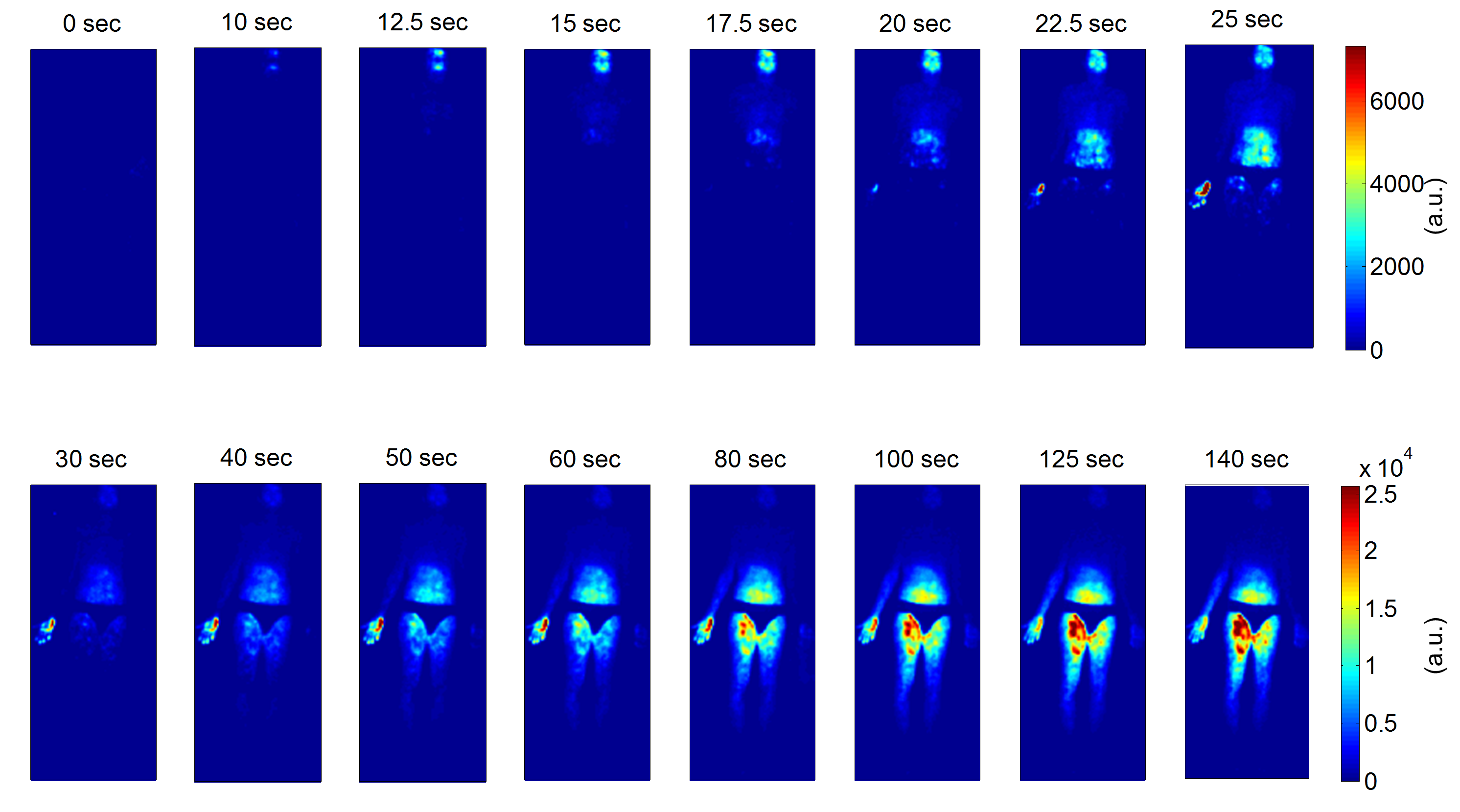

Supplement: Figure S2 — Whole-body fluorescence image time series of measurement 1 without illumination correction. Time zero seconds is set to the beginning of ICG injection. Time intervals between neighboring images differ as indicated by image headers. Color coding of fluorescence intensity in arbitrary units (a.u.) is equally scaled for neighboring images as indicated by the color bars on the right. (TIF) [file pone.0083749.s002.tif]
